# Arachidonic acid promotes myeloid differentiation of splenic CD45- Ter119+ cells in myeloproliferative neoplasm

**DOI:** 10.7150/jca.110478

**Published:** 2025-03-31

**Authors:** Linlin Zhang, Yi Yang, Xiao Yu, Guodong Li, Peihua Zhang, Xiaomin Ren, Siyu Luo, Lin Li, Gustave Munyurangabo, Yachun Jia, Lingqin Song, Aili He, Guangyao Kong

**Affiliations:** 1 Department of Hematology, The Second Affiliated Hospital of Xi'an Jiaotong University, Xi'an, Shaanxi, P. R. China.; 2 Precision Medical Institute, The Second Affiliated Hospital of Xi'an Jiaotong University, Xi'an, Shaanxi, P. R. China.; 3 National and Local Joint Engineering Research Center of Biodiagnosis and Biotherapy, The Second Affiliated Hospital of Xi'an Jiaotong University, Xi'an, Shaanxi, P. R. China.; 4 Department of Oncology, The Second Affiliated Hospital of Xi'an Jiaotong University, Xi'an, Shaanxi, P. R. China.

**Keywords:** myeloproliferative neoplasm, arachidonic acid, myeloid differentiation, juvenile myelomonocytic leukemia

## Abstract

Although splenic CD45^-^Ter119^+^ cells promote cancer progression by secreting artemin, it remains unclear whether these cells play an important role in myeloproliferative neoplasm (MPN). Here, using a Kras^G12D/+^-induced mouse model of MPN, we demonstrated that the number and cycling of CD45^-^Ter119^+^ cells increased in the spleens of MPN mice. Moreover, these cells could differentiate into myeloid cells upon stimulation with GM-CSF and mIL-6. Through RNA sequencing, we further revealed that myeloid genes, such as Hoxa9, Mpo and Ms4a3, were highly expressed in CD45^-^Ter119^+^ cells. Mechanistically, we showed that the arachidonic acid content was significantly elevated in splenic CD45^-^Ter119^+^ cells, and exogenous arachidonic acid mediated the differentiation of splenic CD45^-^Ter119^+^ cells into myeloid cells. Our results revealed that splenic CD45^-^Ter119^+^ cells play a crucial role in myeloid leukemia and that arachidonic acid could be a potential therapeutic target for MPN treatment.

## Introduction

Myeloproliferative neoplasm (MPN) is a clonal hematological malignancy caused by the abnormal differentiation of hematopoietic stem cells into one or more myeloid lineages^1^. According to the 5^th^ edition of the WHO classification of hematolymphoid tumors, juvenile myelomonocytic leukemia (JMML) is a myeloproliferative neoplasm of early childhood in which RAS pathway is activated^2^. JMML is characterized by bone marrow hematopoietic progenitor cell hypersensitivity to granulocyte‒macrophage colony-stimulating factor and the excessive production of granulocytes and monocytes^3^. Previous studies have proven that Kras^G12D/+^ mutation induces a JMML-like MPN and has been recognized as a prevalent mutation in JMML patients^4^,^5^. MPN is usually rapidly fatal due to multiorgan failure or progression toward acute myeloid leukemia unless allogeneic hematopoietic stem cell transplantation (HSCT) is performed^6^,^7^. However, there is a significant risk of post-HSCT recurrence, and the overall survival is only 50-60%^8^,^9^. Therefore, there is an urgent need to identify new therapeutic targets.

Metabolism can strongly affect tumor cell growth and their drug sensitivity. Therefore, revealing the metabolic characteristics and the underlying regulatory mechanisms of splenic CD45^-^Ter119^+^ cells may be important for disease management. In particular, arachidonic acid metabolism plays a crucial role in many kinds of tumors. For example, arachidonic acid metabolism controls alternative macrophage activation by regulating oxidative phosphorylation in a PPARγ-dependent manner^10^. The FADS1-AA-PGE2 axis promotes the development of colorectal cancer by regulating the intestinal microecology of gram-negative bacteria^11^. However, the role of arachidonic acid in MPNs remains unexplored.

A prior study suggested that splenic Ter-cells(CD45^-^Ter119^+^CD71^+^ cells) in animals with advanced solid tumors secrete artemin (ARTN) to promote tumor progression^12^. Splenomegaly is the main clinical symptom of MPN patients. However, almost all previous studies have focused on the bone marrow and peripheral blood of MPN patients, and the role of an enlarged spleen in the occurrence and development of MPNs has not been reported. Here, we found that the number of CD45^-^Ter119^+^ cells increased in the enlarged spleen of MPN model mice and that arachidonic acid produced by tumor cells promoted the differentiation of CD45^-^Ter119^+^ cells into myeloid cells.

## Methods

### Mice

The mice were bred under specific pathogen-free (SPF) conditions, and the mouse experiments were approved by the ethics committee of the Second Affiliated Hospital of Xi'an Jiaotong University.

### DNA extraction and genotyping

The tails from 4-week-old mice were cut and digested according to reported procedures. The samples were subsequently centrifuged, and the DNA was placed on ice as a template for PCR. PCR genotyping of *Mx1-Cre* mice was performed on the mouse tail DNA using a cocktail of two primers (forward: GAACCTGAAGATGTTCGCGAT; reverse:

ACCGTCAGTACGTGAGATATC), and the PCR conditions were as follows: 94 °C for 5 minutes, followed by 35 cycles of 94 °C for 20 s, 60 °C for 30 s, and 72 °C for 50 s, with a final extension at 72 °C for 5 minutes. PCR genotyping of *Kras^G12D/+^* mice was performed on the mouse tail DNA using a cocktail of two primers (wild-type: TGTCTTTCCCCAGCACAGT; mutant: GCAGGTCGAGGGACCTAATA), and the cycling conditions were as follows: 94 °C for 3 minutes, followed by 34 cycles of 94 °C for 30 s, 69 °C for 1 minute, and 72 °C for 1 minute, with a final extension at 72 °C for 2 minutes.

### ELISA

Splenic CD45^-^ cells were selected by MACS and suspended in phosphate-buffered saline (PBS). Serum (top phase) was collected after centrifugation at 1000 × g for 20 minutes. The concentration of arachidonic acid in the serum was measured with an arachidonic acid ELISA kit (CEB098Ge for pan-species, Cloud-Clone Corp).

### Real-time PCR

Total RNA from splenic CD45^-^ cells was isolated by RNAiso Easy according to the manufacturer's instructions (TaKaRa). cDNA was synthesized from RNA via reverse transcription using the PrimeScript RT reagent Kit with gDNA Eraser (TaKaRa, AN11362A). The real-time PCR kit (TB Green Premix Ex Taq, AM43162A) was purchased from Takara Bio, Inc. PCR was performed using a QuantStudio system (Thermo). The primer sequences are listed in Table S1. Target gene expression was normalized to that of GAPDH using the 2^-∆∆Ct^ method.

### Cell cycle assays

The cell cycle distribution was analyzed using Ki67-PE and DAPI-N450. First, all the samples were resuspended in 100 μL of 2% FBS in PBS, and 100 μL of 4% PFA/PBS was added. Then, the samples were incubated in the dark at room temperature for 10 min. After washing once with 1 mL of 2% FBS in PBS, all the samples were resuspended in 100 μL of 0.1% saponin in PBS, and 5 μL of PE-conjugated anti-KI-67 was added. All the samples were subsequently incubated in the dark at room temperature for 45 min. Then, the cells were washed once with 1 mL of 2% FBS in PBS, after which 200 μL of DAPI solution (in 2% FBS in PBS) was added. Finally, the samples were incubated in the dark for 1 hour on ice or at 4 °C overnight before flow cytometry analysis. The results were analyzed with Beckman software.

### Apoptosis assays

Apoptosis was detected with Annexin V-PE/Red Nucleus II-APC (UElandy, Suzhou, China). Briefly, MACS-sorted splenic CD45- cells were resuspended in 100 μL of binding buffer, 5 μL of Annexin V-PE and 5 μL of Red Nucleus II were added, and the mixture was incubated at room temperature for 20 minutes in the dark. Apoptosis was then assessed via flow cytometry, and the results were analyzed with Beckman software.

### Bioinformatics analysis

Transcriptome sequencing and analysis were conducted by OE Biotech Co., Ltd. (Shanghai, China). The raw data were processed using Trimmomatic^13^. The poly-N and low-quality reads were removed to obtain the clean reads. The clean reads were subsequently mapped to the reference genome using HISAT2^14^. The FPKM^15^ value of each gene was calculated using Cufflinks^16^, and the read counts of each gene were obtained with htseq-count^17^. DEGs were identified using the DESeq R package functions estimateSizeFactors and nbinomTest. A P value < 0.05 and a fold change >2 or < 0.5 were the criteria for identifying the DEGs. Hierarchical cluster analysis of the DEGs was performed to explore gene expression patterns. GO enrichment and KEGG pathway enrichment analyses of the DEGs were performed using R on the basis of the hypergeometric distribution^18^.

Widely targeted metabolomics sequencing and analysis were performed by MetWare Biotechnology Co., Ltd. (Wuhan, China). Unsupervised PCA (principal component analysis) was performed using the statistics function prcomp within R (www.r-project.org). The data were subjected to unit variance scaling before unsupervised PCA. Significantly differentially abundant metabolites between groups were determined by VIP ≥ 1 and absolute Log2FC (fold change) ≥ 1. The VIP values were extracted from the OPLS-DA results, which were also used to generate score plots and permutation plots, using the R package MetaboAnalystR. The data were log transformed (log2) and mean centered before OPLS-DA. To avoid overfitting, a permutation test with 200 permutations was performed. KEGG analysis was performed using OmicShare tools, a free online platform for data analysis (http://www.omicshare.com/tools).

### Quantification and statistical analysis

No statistical methods were used to predetermine sample size. All the data were analyzed using an unpaired Student's t test. Statistical analyses were performed using GraphPad Prism 8.0.2 software (GraphPad Software, Inc.). P values of less than 0.05 were considered to indicate statistical significance. In the figures, asterisks indicate significant differences, as follows: *p < 0.05, **p < 0.01, ***p < 0.001, and ****p < 0.0001.

## Results

### The number of CD45^-^Ter119^+^ cells increases in the enlarged spleen with MPN

**Figure 1:**
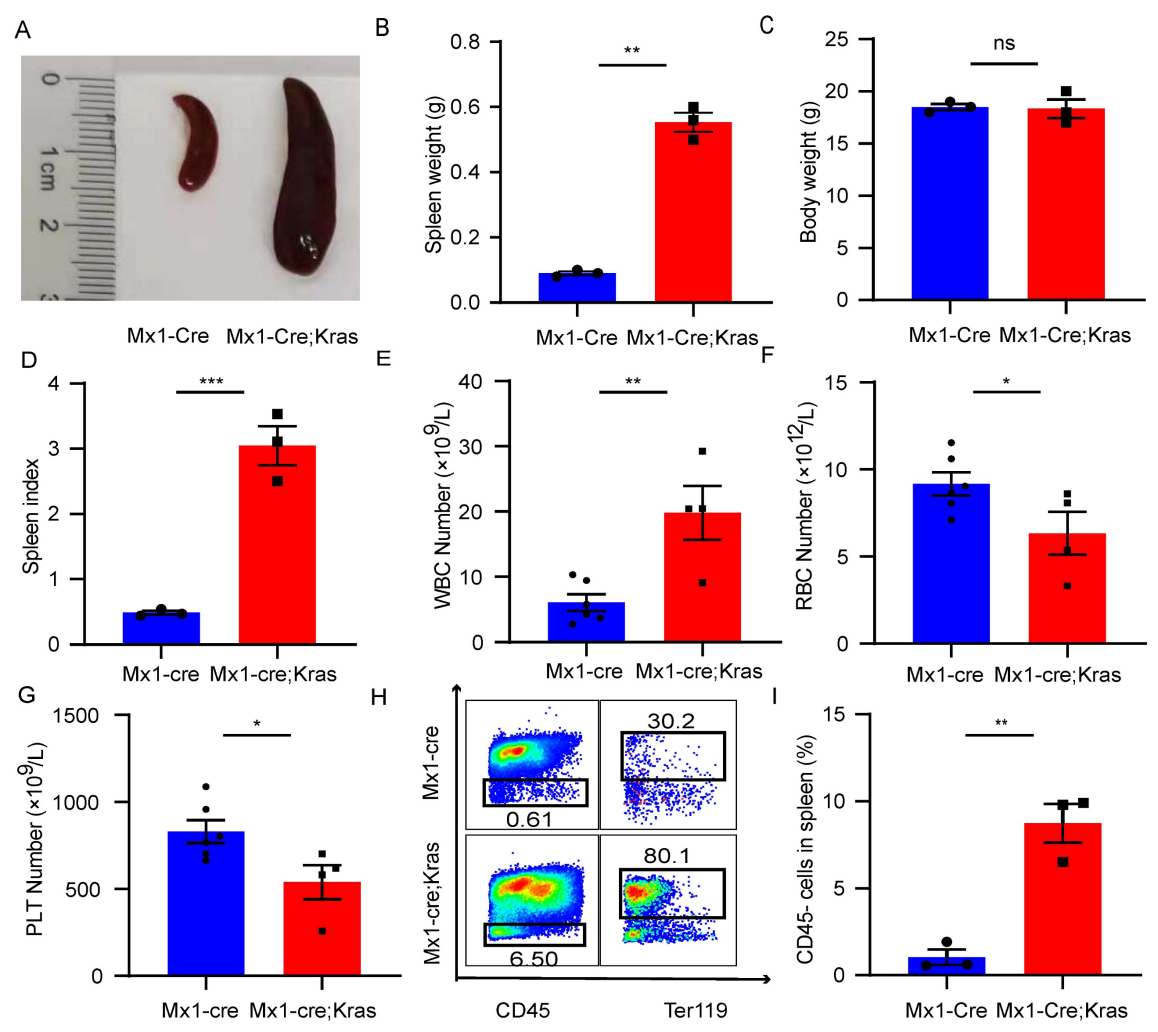
** The number of CD45^-^Ter119^+^ cells increases in the enlarged spleens of MPN model mice.** (A) Size of the spleens from wild-type mice and Mx1-Cre;Kras^G12D/+^ mice. (B-D) Spleen weights, body weights and spleen indices. The data are presented as the means ± SEMs from 3 independent experiments. (E-G) Numbers of WBCs, RBCs and platelets in the peripheral blood. The numbers of wild-type mice and Mx1-Cre;Kras^G12D/+^ mice were 6 and 4, respectively. The data are presented as the means ± SEMs. (H) Flow cytometry analysis of CD45-Ter119+ cells in the spleen. (I) Quantification of CD45-Ter119+ cells in the spleen. The data are presented as the means ± SEMs from 3 independent experiments.

To explore the role of CD45^-^Ter119^+^ cells in the enlarged spleen with MPNs, we established a murine MPN model by crossing Mx1-Cre mice and Kras^G12D/+^ mice. Conditional activation of the constitutively active oncogene Kras^ G12D/+^ in mouse hematopoietic cells promotes monocytosis, which mimics many characteristics of MPN patients. To induce Cre expression, 7-week-old mice were injected intraperitoneally with 5 μg/g polyinosine polycytidylic acid every other day for a total of 2 doses beginning at exactly seven weeks of age. The mice were sacrificed at exactly eight weeks of age for analysis. Morphologically, the spleens of Mx1-Cre; Kras^ G12D/+^ mice (CK) was larger than that of normal mice (Figure 1A). Although there was a significant difference in spleen weight between these two groups, there was no difference in body weight (Figure 1B and 1C). However, the spleen index of the CK mice was greater than that of normal mice (Figure 1D). Consistent with previous results, the CK mice presented increased leukocyte cell counts, decreased red blood cell counts and decreased platelet counts in the peripheral blood (Figure 1E-1G). After lysis and removal of the erythrocytes using red blood cell lysis buffer, we examined the expression of CD45 in the enlarged spleens. In MPN mice, approximately 10% of the splenocytes were CD45^-^, while approximately 1% of the splenocytes were CD45^-^ in the normal mice (Figure 1H-1I). Consistent with prior research, these cells also expressed CD71. The results revealed that the number of CD45^-^Ter119^+^ cells increased in the enlarged spleens of MPN mice.

### Splenic CD45^-^Ter119^+^ cells are able to differentiate into myeloid cells in vitro

**Figure 2:**
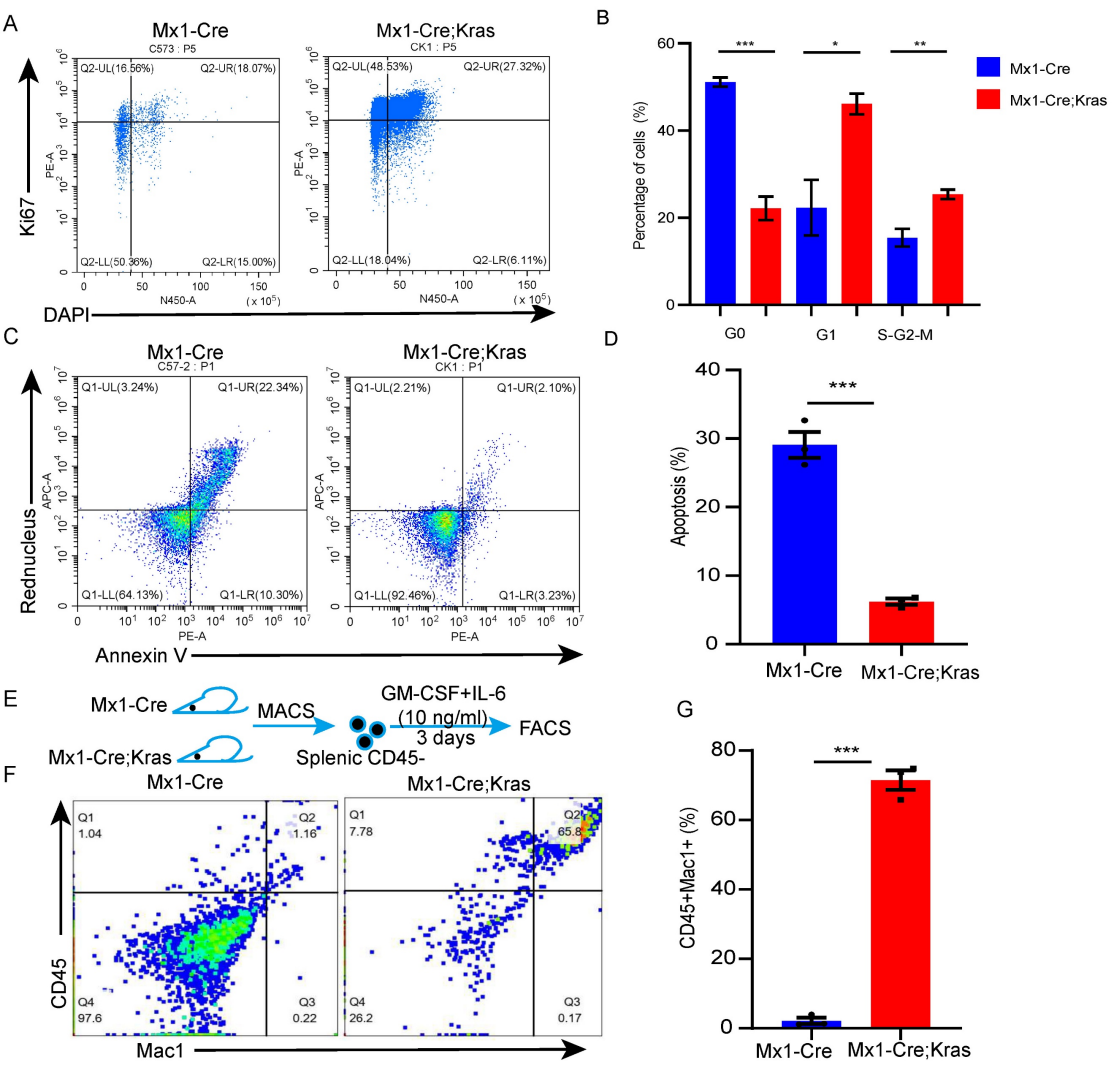
** Splenic CD45^-^Ter119^+^ cells can differentiate into myeloid cells in vitro.** (A-B) Cell cycle analysis of splenic CD45^-^ cells using Ki67 and DAPI. (C-D) Apoptosis of splenic CD45^-^ cells assessed via Annexin V and Red Nucleus II. (E-G) Splenic CD45^-^ cells were isolated from wild-type and Mx1-Cre;Kras^G12D/+^ mice and cultured with GM-CSF and mIL-6 for 3 days (n=3 per group). The data are presented as the means ± SEMs from 3 independent experiments.

To better understand the biology of CD45^-^Ter119^+^ cells, we conducted cell cycle and cell apoptosis assays. Cell cycle analysis verified that the number of CK splenic CD45^-^Ter119^+^ cells underwent more cycling than the wild-type cells did (Figure 2A-2B). The results of the cell apoptosis assay revealed that the percentage of apoptotic CK splenic CD45^-^ cells was lower than that of wild-type cells (Figure 2C-2D). CD45^+^ EPCs from cancer patients and tumor-bearing mice can be induced to differentiate into myeloid cells 19; therefore, to determine whether these cells in mice with MPNs have myeloid differentiation potential, we purified CD45^-^Ter119^+^ cells from the spleens of MPN mice and cultured them in the presence of GM-CSF and mIL-6 in vitro 20. Approximately 67.8% of the cells collected from the enlarged spleens of MPN mice were CD45^+^Mac1^+^, whereas none of the CD45^-^Ter119^+^ cells collected from wild-type mice differentiated into myeloid cells (Figure 2E-2G). Together, these data suggest that there are more splenic CD45^-^Ter119^+^ cells in MPN mice and these cells can differentiate into myeloid cells.

### Splenic CD45^-^ cells express myeloid-related genes

**Figure 3:**
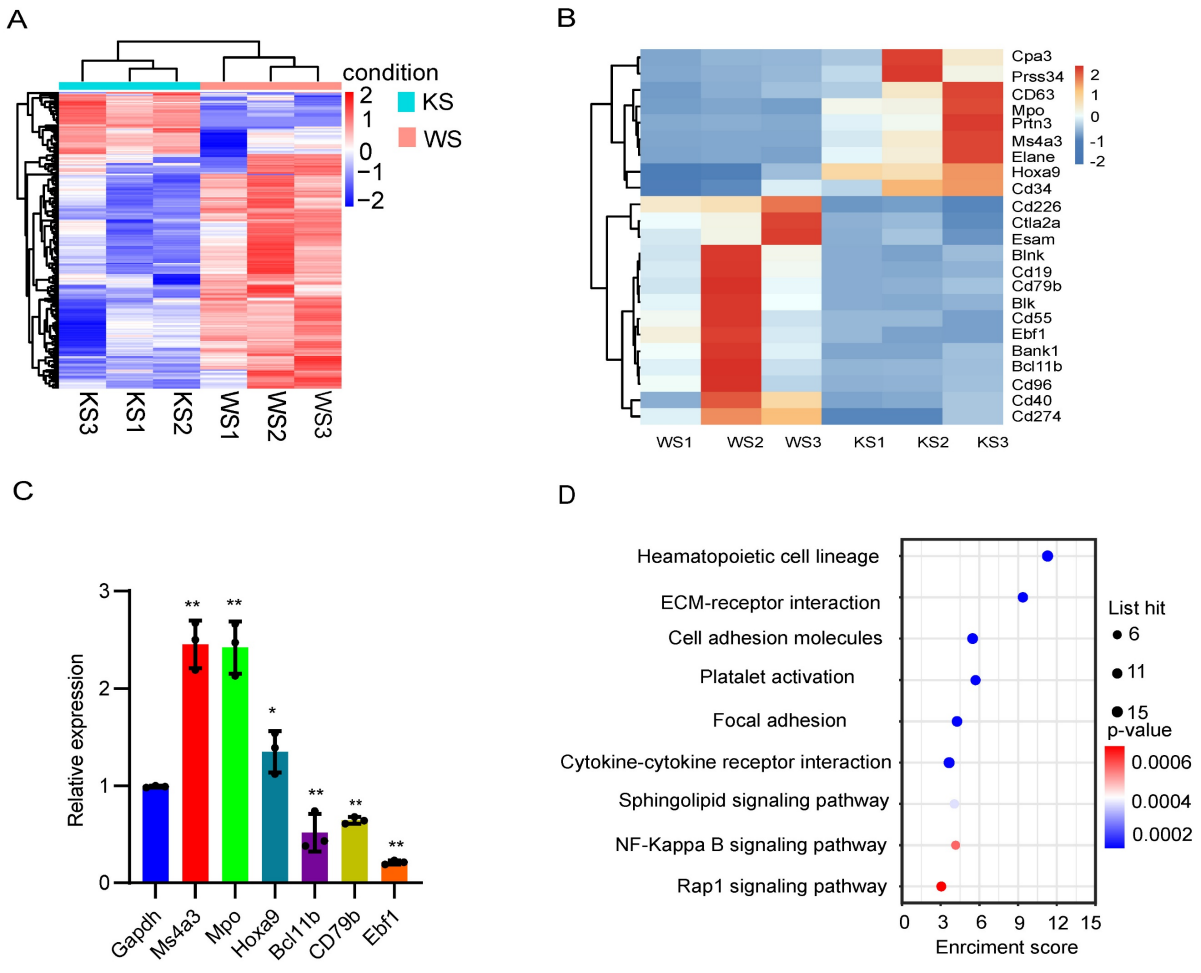
** Splenic CD45^-^ cells express myeloid-related genes.** (A) Heatmap of the RNA-seq data of splenic CD45^-^ cells. (B) Differentially expressed genes between wild-type and Mx1-Cre;Kras^G12D/+^ CD45^-^ cells. (C) The expression of the differentially expressed genes was verified by qPCR. (D) KEGG pathway analysis of differentially expressed genes.

To assess the transcriptional characteristics of splenic CD45^-^ cells in MPN model mice, we collected spleen cells from MPN and wild-type mice and then purified the CD45^-^ cells for bulk RNA-seq. Hereinafter, the KS group refers to group of Mx1-Cre;Kras mice, and the WS group refers to the group of normal wild-type mice. First, principal component analysis (PCA) of the relative abundances of genes revealed significant separation between the KS group and WS group using the first two principal component scores of PC1 and PC2, which explained 63.13% and 22.78% of the variance, respectively (Figure S1A). We subsequently identified 300 differentially expressed genes, among which 63 were upregulated and 237 were downregulated (fold change ≥ 2, p ≤ 0.05; Figure 3A). A heatmap was constructed to analyze the significantly differentially expressed genes, and the differential expression matrix verified that the differentially expressed genes in the KS cluster, such as Hoxa9, Ma4a3 and Mpo, were characteristic of myeloid cells (Figure 3B). qRT‒PCR confirmed that myeloid-related genes were significantly upregulated and that lymphoid-related genes were downregulated in MPN mice (Figure 3C). KEGG analysis revealed that CK CD45^-^ cells were enriched in genes involved in the hematopoietic cell lineage and the sphingolipid signaling pathway (Figure 3D). PPI analysis revealed that many genes related to myeloid cells were highly expressed in MPN mice, whereas genes related to lymphoid cells and platelets were downregulated (Figure S1B). These data reveal that splenic CD45^-^ cells from MPN mice express myeloid-related genes.

### The level of arachidonic acid is elevated in splenic CD45^-^ cells

**Figure 4:**
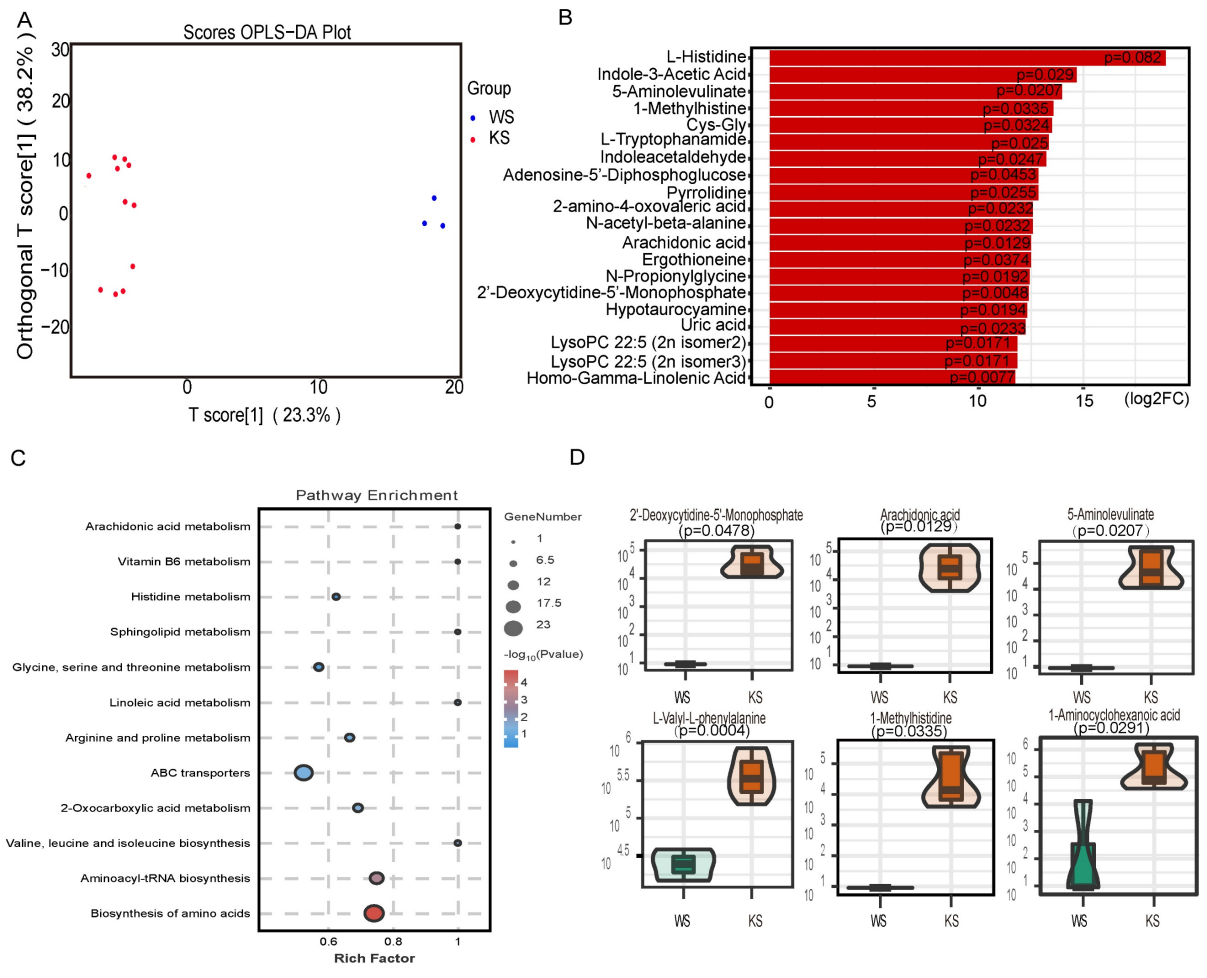
** The arachidonic acid content is elevated in splenic CD45^-^ cells.** (A) OPLS-DA of splenic CD45^-^ cells. (B) Fold changes in the abundances of the top 20 differentially abundant metabolites. (C) KEGG pathway analysis of the differentially abundant metabolites. (D) Relative amounts of different metabolites in splenic CD45^-^ cells.

With the development of metabolomics, an increasing number of studies have shown that metabonomics may play an important role in disease. To explore the factor that promotes lineage differentiation, we used LC‒MS for nontargeted metabolomics analysis to examine the metabolome of CD45^-^ cells. Because of cell number limitations, we isolated CD45^-^ cells by magnetic-activated cell sorting for these studies. OPLS-DA revealed that the experimental group and the control group were clearly separated (Figure 4A). We identified 162 metabolites with significantly different abundances. Figure 4B shows the top 20 significantly differentially abundant metabolites. KEGG analysis revealed that certain pathways, including arachidonic acid metabolism, sphingolipid metabolism and linoleic acid metabolism, were enriched in the differentially abundant metabolites in CK splenic CD45- cells (Figure 4C). The violin plot revealed that the arachidonic acid content in the CK group was greater than that in the control group (Figure 4D). Thus, these data indicate that CK splenic CD45^-^ cells exhibit distinct metabolism differences and that lineage differentiation may be mediated by the elevated level of arachidonic acid.

### Arachidonic acid mediates the differentiation of splenic CD45-Ter119+ cells into myeloid cells

**Figure 5:**
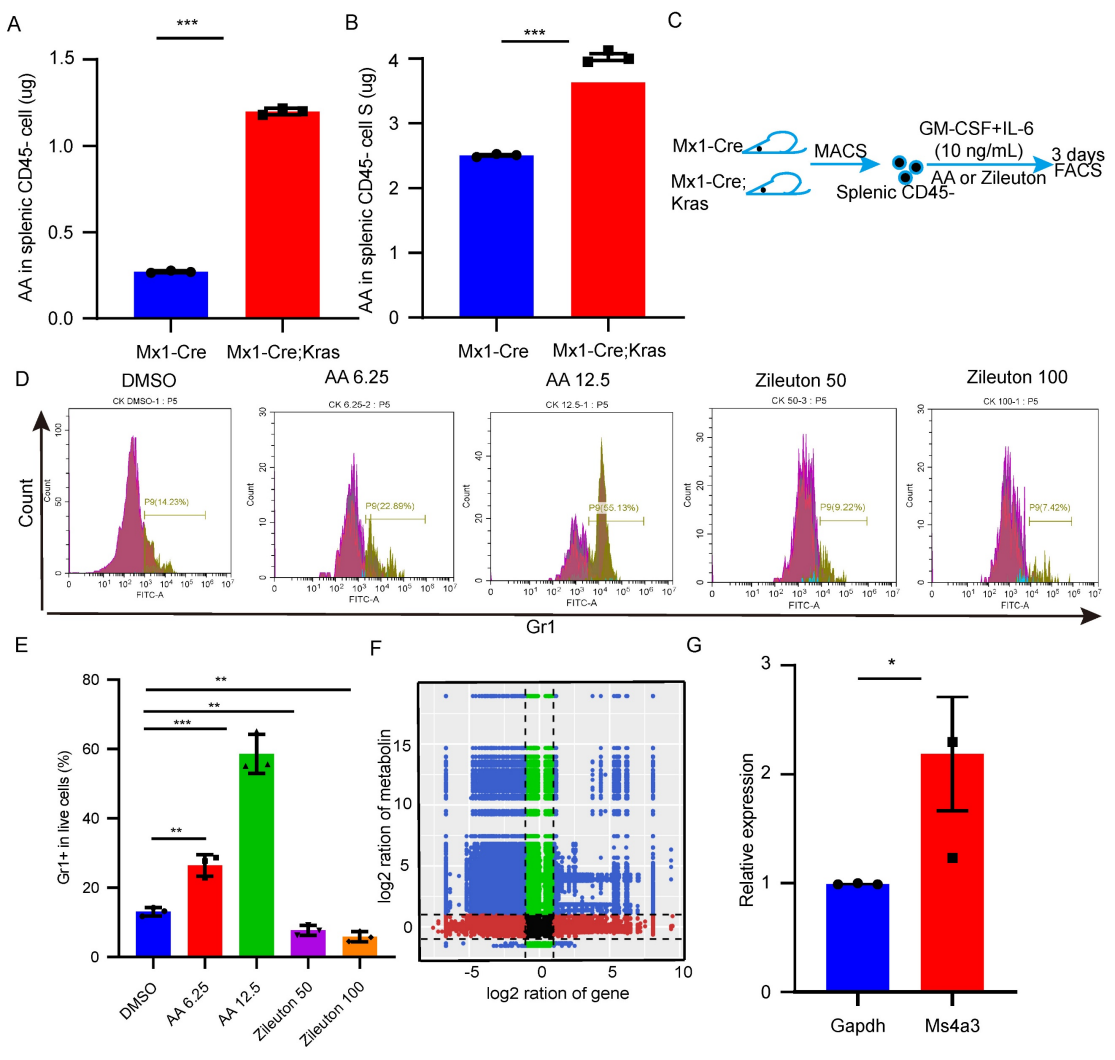
** Arachidonic acid mediates the differentiation of splenic CD45^-^Ter119^+^ cells into myeloid cells.** (A-B) The content of arachidonic acid in splenic CD45^-^ cells and the supernatant was determined with an ELISA kit. (C-E) CD45^-^ cells were isolated and cultured in vitro in the presence of arachidonic acid and zileuton for 3 days. Gr1^+^ cells were detected by flow cytometry. The data are presented as the means ± SEMs from 3 independent experiments. (F) Analysis of the nine quadrants showed that the arachidonic acid content was related to myeloid gene expression. (G) qPCR verified the expression of myeloid genes after 3 days of culture.

To further investigate the mechanism underlying differentiation, we found that arachidonic acid was more abundant in CK splenic CD45^-^ cells than in the splenic CD45^-^ cells from the other groups. Then, we used an ELISA kit to confirm the content of arachidonic acid (Figure 5A-B). Thus, we hypothesized that arachidonic acid might be important for the differentiation of CD45^-^ cells into myeloid cells. To determine whether arachidonic acid plays a functional role in splenic CD45^-^ cell differentiation, we treated CK splenic CD45- cells with different concentrations of arachidonic acid and zileuton (Figure 5C-D). As shown in Figure 5E, arachidonic acid significantly increased the number of Gr1+ cells, whereas the addition of zileuton caused a dose-dependent reduction in the number of these cells. Taken together, these data demonstrated that arachidonic acid might trigger splenic CD45^-^ cell differentiation. To determine how arachidonic acid can promote splenic CD45^-^ cell differentiation, we performed a combined transcriptomics and metabolomics analysis. Correlation analysis of the nine quadrants of the scatterplot revealed that arachidonic acid was positively correlated with approximately 137 genes, including myeloid-specific genes such as Hoxa9, Mpo and Ms4a3 (Figure 5F). qPCR revealed that Ms4a3 was highly expressed in culture (Figure 5G). Therefore, these results indicate that arachidonic acid mediates the differentiation of CK splenic CD45^-^Ter119^+^ cells into myeloid cells by upregulating these myeloid-related genes.

## Discussion

Despite extensive research, the survival rate of MPN patients remains poor^21^. While many studies have focused on intrinsic and extrinsic cell factors, few studies have focused on the roles of extramedullary organs in MPN. Splenomegaly is the main symptom of MPN, but the role of the spleen in MPN progression has not yet been reported. Immune cells (all CD45^+^) play important roles in disease progression; however, nonleukocyte cells can also promote tumor progression. Our results demonstrate a novel role for splenic CD45^-^Ter119^+^ cells in MPN. First, we showed that the number of CD45^-^ cells, most of which express Ter119, was increased in the spleens of CK mice, as shown in a previous study. Second, we found that myeloid gene expression was positively correlated with the arachidonic acid content. Nontargeted metabolomics analysis revealed that CK spleen cells can produce a large amount of arachidonic acid. After treating CD45^-^ cells with arachidonic acid, these cells can differentiate into CD45^+^Mac1^+^ cells. Third, splenic CD45^-^ cells are induced by arachidonic acid to upregulate myeloid genes and therefore obtain the ability to differentiate into myeloid cells in vitro. Taken together, these findings support a model in which splenic CD45^-^Ter119^+^ cells are educated by the niche to acquire unique metabolic properties that promote differentiation and disease progression.

Recent studies have verified that tumors can induce erythroid lineage cells to differentiate into tumor-associated myeloid cells via GM-CSF signaling; however, these studies did not consider metabolic factors, which may play important roles in tumor progression. For example, the liver microenvironment triggers metabolic changes by increasing the expression of endothelial lipase (LIPG) in leukemia cells. This not only promotes tumor cell growth through PUFA-mediated pathways but also enhances their survival by inducing antiapoptotic protein expression^22^. In this study, we found that arachidonic acid can promote the lineage differentiation of spleen cells. However, because mice are not easy to obtain, we did not determine whether arachidonic acid from the spleen can promote the progression of MPN by affecting the function of bone marrow hematopoietic stem cells.

Arachidonic acid is an ω-6 polyunsaturated fatty acid that can be metabolized by three enzyme systems, cyclooxygenases, lipoxygenases and cytochrome P450 enzymes^23^,^24^. However, enolase 1, an RNA-binding protein, binds and stabilizes YAP1 mRNA to promote liver cancer by activating arachidonic acid metabolism in cells 25. Arachidonic acid and its metabolites have stimulated widespread interest in inflammatory disease and cancer research. In combination with IFN-γ derived from CD8^+^ T cells, arachidonic acid induces tumor cell ferroptosis in an ACLS4-dependent manner 26. Cyclooxygenases metabolize arachidonic acid to PGE2, and PGE2-EP2/EP4 inhibits TIL function and promotes immune escape by interfering with the IL-2 signaling pathway, which provides new targets and strategies for immunotherapy^27^,^28^.

In summary, our findings demonstrate that CD45^-^Ter119^+^ cells exist in the enlarged spleen of individuals with MPN and that they have unique transcriptomic and metabolic properties. Moreover, arachidonic acid plays a role in myeloid differentiation. We therefore hypothesize that designing future MPN therapies to address the influence of the enlarged spleen may improve therapeutic outcomes. A limitations of our study are that we did not elucidate the source of arachidonic acid and the role of its metabolites in hematopoietic stem cells, which need further investigation.

## Supplementary Material

Supplementary figure and table.
