# Supplementary material for: Arachidonic acid promotes myeloid differentiation of splenic CD45- Ter119+ cells in myeloproliferative neoplasm
Source: J Cancer. 2025 Mar 31;16(7):2289–97. doi: 10.7150/jca.110478 (PMC12036098; doi:10.7150/jca.110478)
Supplement: Supplementary file 1 — Supplementary figure and table. [file jcav16p2289s1.pdf]

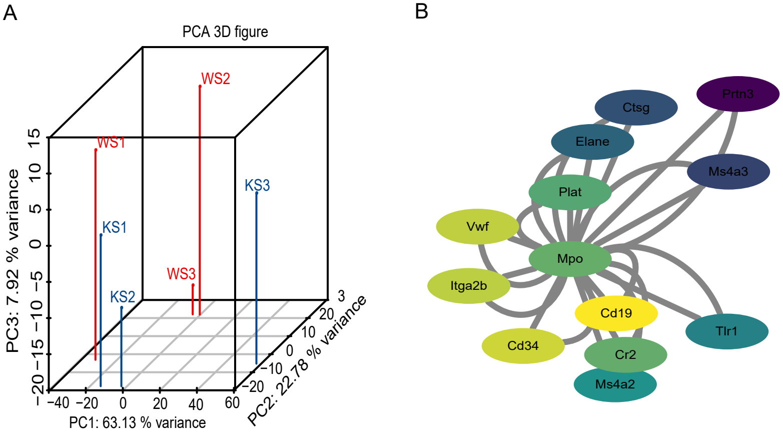
A
PCA 3D figure
WS1
WS2
WS3
KS1
KS2
KS3
PC1: 63.13 % variance
PC2: 22.78 % variance
PC3: 7.92 % variance
B
Prtn3
Ctsg
Elane
Ms4a3
Plat
Vwf
Mpo
Itga2b
Cd19
Tlr1
Cd34
Cr2
Ms4a2

Figure S1 RNA-seq analysis of splenic CD45- cells.

(A) PCA analysis of splenic CD45- cells from MPN and wild type mice.

(B) PPI interaction network of myeloid related genes and lymphoid related genes.

Supplementary table1 RT-qPCR primer sequences for mouse genes

Name	Sequence 5'-3'
Hoxa9-F	CCCTGACTGACTATGCTTGTG
Hoxa9-R	GCATCGCTTCTTCCGAGTG
Ms4a3-F	GTGGTTCTGTTTATCAGCCCTT
Ms4a3-R	ACAGTGGGTAGCCTGTGTAGA
Mpo-F	AGTTGTGCTGAGCTGTATGGA
Mpo-R	CGGCTGCTTGAAGTAAAACAGG
Cd79b-F	CGAGGTTTGCAGCCAAAAAG
Cd79b-R	CACAATGCGTCCCTCTTCTG
Ebf1-F	GCATCCAACGGAGTGGAAG
Ebf1-R	GATTTCCGCAGGTTAGAAGGC
Bcl11b-F	CCTCCGTGATTACTTCACCTCT
Bcl11b-R	TGACCCTCACCCTGAGTCC
Gapdh-F	AGCTTGTCATCAACGGGAAG
Gapdh-R	TTTGATGTTAGTGGGGTCTCG
